# Liquation Cracking Susceptibility and Mechanical Properties of 7075 Aluminum Alloy GTAW Joints

**DOI:** 10.3390/ma15103651

**Published:** 2022-05-20

**Authors:** Zhuoxin Li, Yulin Zhang, Hong Li, Yipeng Wang, Lijuan Wang, Yu Zhang

**Affiliations:** 1 Institute of Light Alloy and Processing, Faculty of Materials and Manufacturing, Beijing University of Technology, Beijing 100124, China; zhxlee@bjut.edu.cn (Z.L.); zhangyulin_666@163.com (Y.Z.); hongli@bjut.edu.cn (H.L.); wyp@bjut.edu.cn (Y.W.); 2 School of Mechanical and Engineering, Tianjin Sino-German University of Applied Sciences, Tianjin 300350, China; wang_040305@163.com

**Keywords:** aluminum alloy, liquation cracking, gas tungsten arc welding, partial melting zone

## Abstract

In this work, aluminum alloy 7075-T651 was welded by using customized Al-Cu-Si and Al-Cu-Mg-Zn filler wire during gas tungsten arc welding. The liquation cracking susceptibility of the joints was tested under a circular-patch welding experiment. Besides, the temperature vs. solid fraction curves (*T-f_S_*) was calculated for different samples to reveal the formation mechanism of liquation cracking. The joint was susceptible to liquation cracking if (*f_S_*)_weld_ > (*f_S_*)_workpiece_ during the cooling stage. The results of the circular-patch welding experiment show that the liquation cracking susceptibility of the joint by using ER5356, Al-Cu_1.5_-Si_4.5_, Al-Cu_3.0_-Si_2.5_, Al-Cu_4.5_-Si_1.5_, Al-Cu_2.3_-Mg_2.3_-Zn_6.6_ and Al-Cu_2.2_-Mg_2.0_-Zn_7.8_ filler metal is 22.8%, 8.3%, 2.8%, 2.8%, 3.3% and 1.4%, respectively. The mechanical test shows that the data dispersion of the 7075 gas tungsten arc welding joint can be decreased by eliminating the liquation crack.

## 1. Introduction

Aluminum alloy was characterized by low-density, corrosion-proof and high-impact energy absorptivity, which led to its extensive application in automotive, marine and aircraft industries [1,2,3]. The high thermal diffusion coefficient of the aluminum alloy makes it is difficult to be effectively heated during the fusion welding process in which case a concentrated heat source is required [4,5]. Besides, to meet the demands of effective heating and oxide film cleaning, an alternating or variable polarity power source is typically used in aluminum alloy arc welding [6,7].

Aluminum alloy 7075 (AA7075) is a kind of high-specific-strength light metal. AA7075 possesses high strength under a peak aged state due to its high mass fraction of the Cu-Mg-Zn alloying element [8]. However, the metallurgical characteristics of AA7075 lead to its high susceptibility to solidification cracking and liquation cracking [9,10]. Finding an optimal combination between the filler wire and base metal to prevent these defects is a major concern in the research field of aluminum alloy arc welding.

Both aluminum and magnesium alloy have a wide temperature gap between liquidus and solidus. During the fusion welding process, a partially melted zone (PMZ) forms adjacent to the fusion zone (FZ) [11,12]. Liquation cracking is a common defect in aluminum or magnesium alloy arc welding joints, which occurs within the PMZ. Kou et al. suggested an effective criterion to predict the liquation cracking susceptibility [10,13]. By considering the dilution rate, the solid fraction (*f_S_*)_weld_ and (*f_S_*)_workpiece_ evolution was calculated within each melting temperature range (*T-f_S_* curve). The joint was susceptible to liquation cracking if (*f_S_*)_weld_ > (*f_S_*)_workpiece_ when this local site was under tension. Adding alloying elements to adjust the T-f_S_ curve of FZ is an effective method to restrain the liquation cracking susceptibility of a welding joint [14]. It is necessary to carry out fundamental research on the cracking phenomenon for metals likely to crack when a custom filler metal was used.

Typically, ER5356 or ER4043 is used as filler wire during the AA7075 arc welding process [10]. The Cu and Zn composition of the FZ is lower than the base metal. This would lead to a decrease in strength and an increase in the susceptibility to galvanic corrosion. Besides, the difference in composition between FZ and PMZ may lead to liquation cracking if (*f_S_*)_weld_ > (*f_S_*)_workpiece_ during the cooling period. It is necessary to develop a novel filler metal for AA7075 arc welding.

In this study, custom Al-Cu-Si and Al-Cu-Mg-Zn filler wires are used in AA7075 gas tungsten arc welding. The liquation cracking susceptibility and mechanical property of the joints are investigated.

## 2. Materials and Methods

### 2.1. Welding Test

Aluminum alloy 7075-T651 (the 0.2% yield strength is 470 MPa while the tensile strength is 540 MPa) was used as a base metal while a series of custom Al-Cu-Si and Al-Cu-Mg-Zn wire was used as filler metal in this study. The main alloying elements of each material are summarized in Table 1.

**Figure 1:**
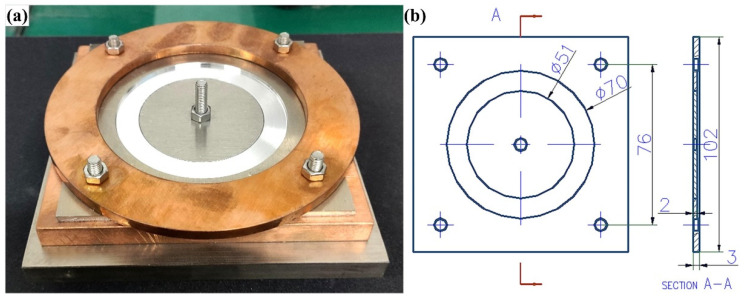
Circular-patch welding experiment: (**a**) Assembly of the parts; (**b**) dimensions of the workpiece (unit: mm).

A 315P AC/DC gas tungsten arc welding machine was used for the welding test. The welding process was conducted under 99.99% high-purity argon shielding gas. The welding current was 85 A under AC mode while the traveling speed of the welding gun was 1 mm/s. The liquation cracking susceptibility for different combinations of the base metal and filler wire was tested by a circular-patch welding experiment of which the samples’ dimensions are shown in Figure 1. After testing, the liquation cracking susceptibility for each joint was recorded by counting the cracking percentage along the outer edge of the circular weld. Besides, the color metallography samples (CM samples) at the interface of FZ/PMZ were prepared to show the cracked or un-cracked morphology (prepared by anodizing using a solution of 25 mL fluoboric acid + 475 mL H_2_O). The anodizing voltage was 20 V, while the anodizing time was 120 s. The sampling position of the CM samples was near the crater of the weld.

**Figure 2:**
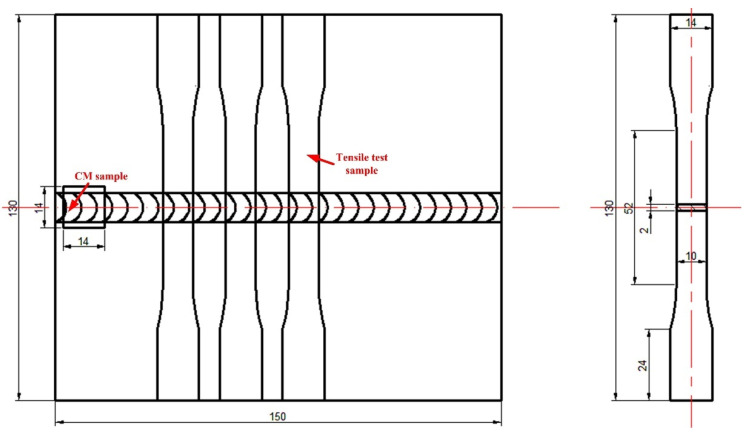
Extracting CM and tensile test samples from the butt-welding joints (unit: mm).

Furthermore, butt-welding joints were prepared and cut for microstructural and tensile test samples, of which the dimensions are shown in Figure 2 (2 mm thickness). The welding parameters were the same as the samples in the circular-patch welding experiment. Moreover, CM samples of the interface of FZ/PMZ in different butt-welded samples were prepared. Electron probe microanalysis (EPMA) for welds using a filler metal of Al-Cu_1.5_-Si_4.5_, Al-Cu_4.5_-Si_1.5_ and Al-Cu_2.3_-Mg_2.3_-Zn_6.6_ was conducted by a Shimadzu EPMA-1720 device. The tensile tests were conducted on a CSS-44100 material test system (three samples repeated) at a tensile speed of 1 mm/min. The fracturing surface was observed by a scanning electron microscope (Hitachi S-3400N).

### 2.2. Liquation Cracking Susceptibility Analysis by T-f_S_ Criterion

**Figure 3:**
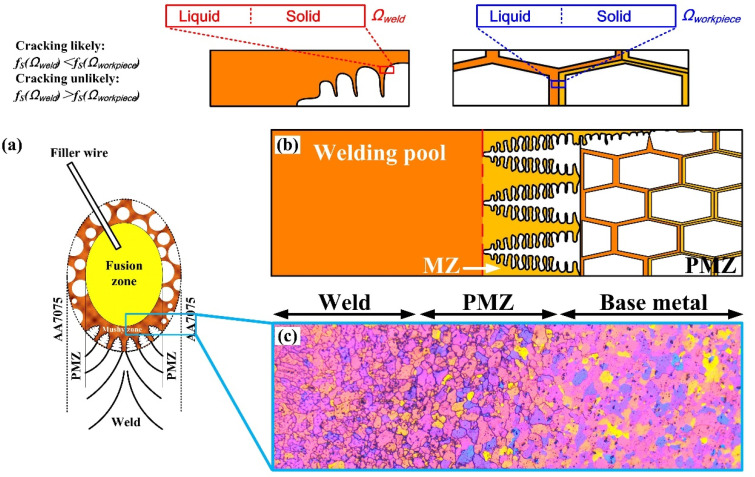
Schematic of analyzing liquation cracking susceptibility by using volume elements Ω: (**a**) Sub-divisions of the welds; (**b**) magnification of the rectangle area in (**a**); (**c**) CM figure of AA7075 GTAW joint of corresponding area.

During AA7075 gas tungsten arc welding (GTAW) with filler wire, the solid–liquid phase transition pattern can be schematically illustrated in Figure 3. There is a thin layer of PMZ in the work piece surrounding the liquid welding pool, while a mushy zone (MZ) exists at the tail zone of the liquid welding pool. A rectangle located at the interface between the MZ and PMZ was enlarged in Figure 3b,c. There is no mass transfer between MZ and PMZ. It is considered that there is a small-volume element Ω within MZ and PMZ. The range of Ω*_weld_* was from the center line of a secondary dendritic arm to its boundary, while the range of Ω*_workpiece_* was from the center line between two residual grains to the solid boundary. *T-f_S_* curves of Ω*_weld_* and Ω*_workpiece_* were calculated. The liquation cracking is susceptible if (*f_S_*)_weld_ > (*f_S_*)_workpiece_ during the cooling stage.

In Figure 3c, the interface between FZ and PMZ can be observed. The MZ forms between the liquidus and solidus of the FZ. So, the MZ/PMZ interface is exhibited at the FZ/PMZ interface at room temperature.

The *T-f_S_* curves of Ω*_weld_* and Ω*_workpiece_* were calculated using JMatPro with an aluminum alloy database. The Scheil–Gulliver solidification model was used. The *T-f_S_* curves could be obtained by using the following equations:

(1) $$\left(C_{i}^{\bar{S}}-C_{i}^{L}\right)df_{L}=f_{L}dC_{i}^{L}$$

(2) $$C_{i}^{\bar{S}}=\sum \left(\frac{f^{j}}{\sum f^{j}}\cdot C_{i}^{j}\right)$$

(3) $$\frac{dC_{i}^{L}}{dC_{i+1}^{L}}=\frac{C_{i}^{L}-C_{i}^{\bar{S}}}{C_{i+1}^{L}-C_{i+1}^{\bar{S}}}$$

where *f_L_* is the liquid fraction while the solid fraction is given by *f_S_ = (1* − *f_L_)*; C is the concentration of a specific element; the superscripts identify the phase, and the subscripts refer to a specific component. By solving the solid fraction evolution within the freezing temperature range of the target materials, the *T-f_S_* curves could be obtained.

The effect of each alloying element (Cu, Mg, Si and Zn) on the solidification path was assessed by calculating the *T-f_S_* curve within the corresponding binary system. The calculation range for each alloying element is from 1 to 10 wt.% with an interval of 1 wt.%. In order to determine the composition of the weld, the dilution of the workpiece is assumed to be 40%. The composition of the AA7075 base metal for *T-f_S_* calculation is 1.5Cu-2.2Mg-0.4Si-5.5Zn (wt.%). Besides, the phase transition of the typical welds during the solidification period was also calculated to aid in the analysis of the microstructure evolution of the AA7075 joint with different filler wires.

## 3. Results and Discussion

### 3.1. Liquation Cracking Susceptibility of Different Base Metal/Filler Wire Combination

By considering the schematic of an aluminum alloy GTAW joint in Figure 3, the relationship between the solidification path and the liquation cracking susceptibility can be illustrated: The temperature field distribution of the joint is continuous. The volume of Ω is infinitely small compared to the welds. So, the temperature of Ω*_weld_* and Ω*_workpiece_* can be considered the same as each other where they are adjacent to the interface between MZ and PMZ. Besides, during the cooling stage, a similar level of tension is generated under both in Ω*_weld_* and Ω*_workpiece_*. Experimental data show the semi-solid Al alloy’s strength increases with the increase in its solid fraction *f_S_* [15]. Under the same temperature and tension, if the *f_S_* in Ω*_weld_* was higher than that of Ω*_workpiece_*, liquation cracking will take place within PMZ, where it is adjacent to MZ.

**Figure 4:**
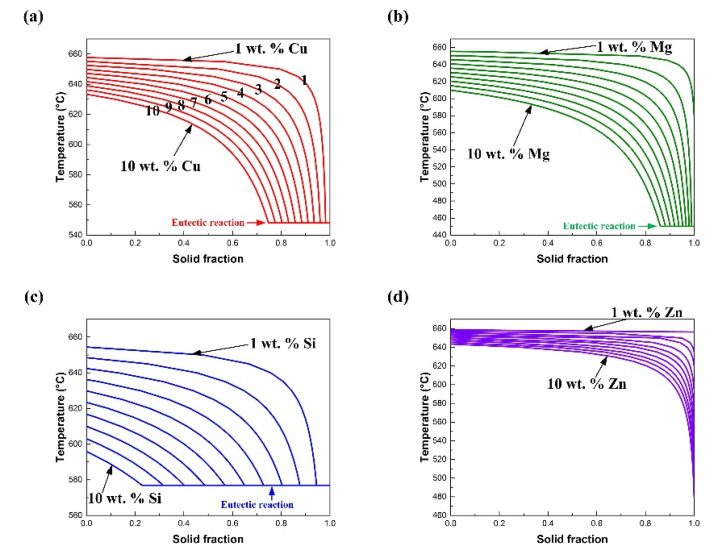
Assessment of each alloying element on the solidification path: (**a**) Al-Cu binary system; (**b**) Al-Mg binary system; (**c**) Al-Si binary system; (**d**) Al-Zn binary system.

Figure 4 shows the effect of the alloying elements on the *T-f_S_* curves. It can be observed that *f_S_* is decreased under a specific temperature by increasing the content of each element. Besides, for Cu, Mg and Si, a eutectic reaction takes place at the end of the solidification process. Typically, the eutectic reaction with intermetallic compounds forms at the interdendritic zone of a weld [6]. This will decrease the ductility of the weld. The assessment of the effects of each alloying element on the solidification path shows that each alloying element shall be added under an appropriate mass fraction in the filler wire. If the content of a specific alloying element is too low, the joint is prone to cracking. If the content of a specific alloying element is too high, the ductility of the weld is compromised.

Table 2 shows the liquation cracking susceptibility of each joint, which is tested by a circular-patch welding experiment. Compared to the welds using ER5356, the liquation cracking susceptibility of welds using Al-Cu-Si and Al-Cu-Mg-Zn filler wire is obviously decreased.

**Figure 5:**
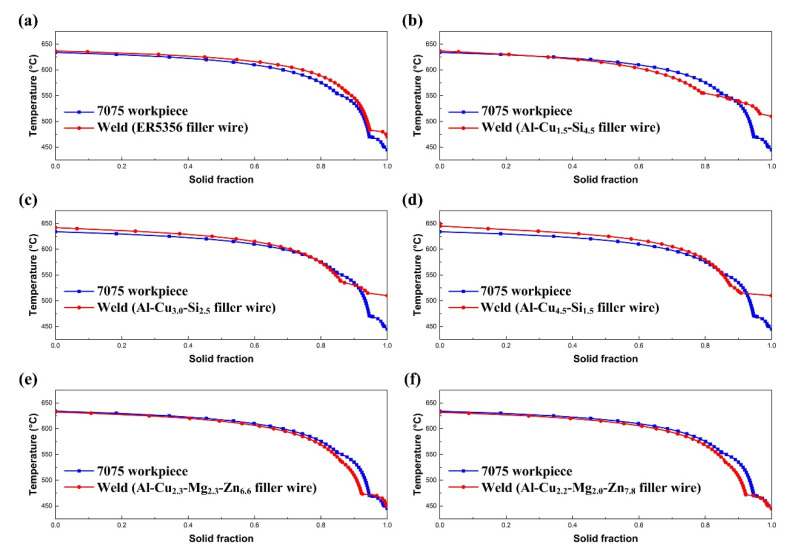
*T-f_S_* curves of different joints: (**a**) The AA7075/ER5356 joint; (**b**) AA7075/Al-Cu_1.5_-Si_4.5_ joint; (**c**) the AA7075/Al-Cu_3.0_-Si_2.5_ joint; (**d**) the AA7075/Al-Cu_4.5_-Si_1.5_ joint; (**e**) the AA7075/Al-Cu_2.3_-Mg_2.3_-Zn_6.6_ joint; (**f**) the AA7075/Al-Cu_2.2_-Mg_2.0_-Zn_7.8_ joint.

Figure 5 shows the *T-f_S_* curves of Ω*_weld_* and Ω*_workpiece_* under different base metal/filler wire combinations. During the entire cooling period, (*f_S_*)_weld_ > (*f_S_*)_workpiece_ for the weld with ER5356. For the weld with Al-Cu-Si wire, (*f_S_*)_weld_ < (*f_S_*)_workpiece_ within the high-temperature zone while (*f_S_*)_weld_ > (*f_S_*)_workpiece_ within the low-temperature zone. For those welds with Al-Cu-Mg-Zn wire, (*f_S_*)_weld_ < (*f_S_*)_workpiece_ within most of the semi-solid temperature range.

For the AA7075/ER5356 joint (Figure 5a), *T-f_S_* curves show that (*f_S_*)_weld_ > (*f_S_*)_workpiece_ at any temperature range, which means the liquation cracking sensibility is high under this base metal/filler wire combination. The literature on magnesium alloy hot cracking shows the crack forms initially at 500 °C [16]. For the AA7075/Al-Cu_1.5_-Si_4.5_ joint, (*f_S_*)_weld_ < (*f_S_*)_workpiece_ within the temperature range of 545~625 °C while (*f_S_*)_weld_ > (*f_S_*)_workpiece_ when the temperature is below 545 °C. So, it is possible that liquation cracking occurs when the FZ/PMZ interface’s temperature decreases below 545 °C. The temperature range of (*f_S_*)_weld_ > (*f_S_*)_workpiece_ for the AA7075/Al-Cu_3.0_-Si_2.5_ joint and the AA7075/Al-Cu_4.5_-Si_1.5_ joint is below 530 °C and 515 °C, respectively. The PMZ’s local strength increases when the local temperature is lowered, which can resist the occurrence of liquation cracking. In fact, the results of the circular-patch welding test in Table 2 show the liquation cracking susceptibility of the AA7075/Al-Cu_1.5_-Si_4.5_ joint is slightly higher than that of the AA7075/Al-Cu_3.0_-Si_2.5_ joint and the AA7075/Al-Cu_4.5_-Si_1.5_ joint. Besides, both *T-f_S_* curves and the circular-patch welding test show that the liquation cracking susceptibility of the AA7075/Al-Cu_2.3_-Mg_2.3_-Zn_6.6_ joint and the AA7075/Al-Cu_2.2_-Mg_2.0_-Zn_7.8_ joint is low.

### 3.2. Liquation Cracking Susceptibility of Different Base Metal/Filler Wire Combinations

**Figure 6:**
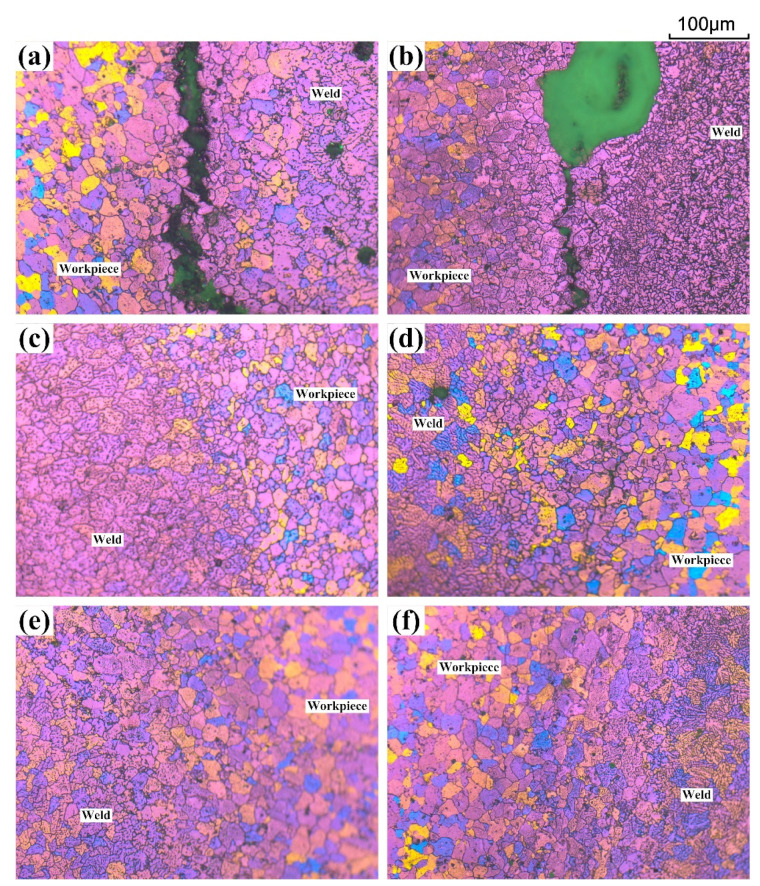
Color metallography images of different joints (circular-patch welding): (**a**) The AA7075/ER5356 joint; (**b**) AA7075/Al-Cu_1.5_-Si_4.5_ joint; (**c**) the AA7075/Al-Cu_3.0_-Si_2.5_ joint; (**d**) the AA7075/Al-Cu_4.5_-Si_1.5_ joint; (**e**) the AA7075/Al-Cu_2.3_-Mg_2.3_-Zn_6.6_ joint; (**f**) the AA7075/Al-Cu_2.2_-Mg_2.0_-Zn_7.8_ joint.

Figure 6 shows the color metallography images of different joints in circular-patch welding tests. It can be observed that the liquation crack appears within the PMZ, adjacent to the FZ, in the AA7075/ER5356 and AA7075/Al-Cu_1.5_-Si_4.5_ joint. By contrast, no liquation crack was found in other samples (near the crater).

**Figure 7:**
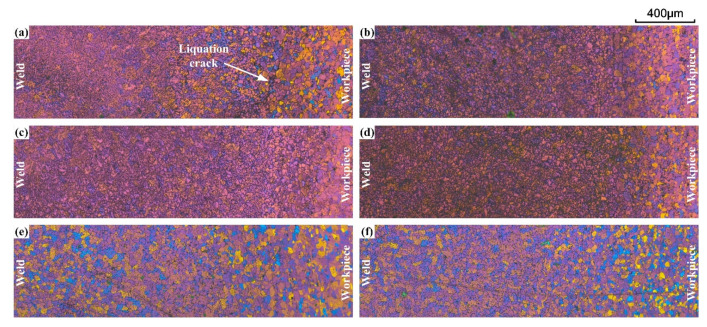
Colur metallography images of different joints (butt-welding): (**a**) The AA7075/ER5356 joint; (**b**) AA7075/Al-Cu_1.5_-Si_4.5_ joint; (**c**) the AA7075/Al-Cu_3.0_-Si_2.5_ joint; (**d**) the AA7075/Al-Cu_4.5_-Si_1.5_ joint; (**e**) the AA7075/Al-Cu_2.3_-Mg_2.3_-Zn_6.6_ joint; (**f**) the AA7075/Al-Cu_2.2_-Mg_2.0_-Zn_7.8_ joint.

Furthermore, liquation cracks could also be observed in the butt-welded sample if the liquation cracking sensibility of the filler/workpiece combination is high (Figure 7). Note that the thermal stress distribution is fairly different between the circular-patch welding sample and the butt-welding sample [17,18]. In the former case, the liquation cracking is prone to occur. In the butt-welding sample, the liquation cracks present as tiny discontinuous cracks. The liquation crack can be observed at the FZ/PMZ interface of the weld with ER5356 filler wire. There is no sign of liquation cracking for other samples.

**Figure 8:**
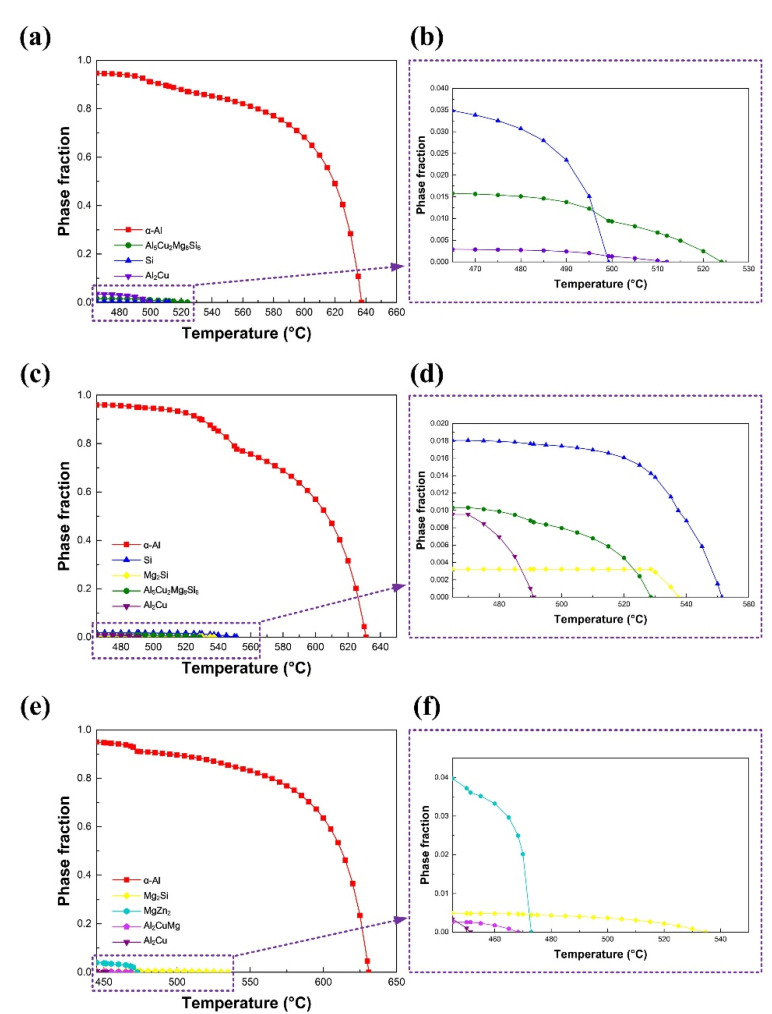
The phase evolution data during solidification process of typical welds: (**a**) The AA7075/Al-Cu4.5-Si1.5 weld; (**b**) the magnification of (**a**); (**c**) the AA7075/Al-Cu1.5-Si4.5 weld; (**d**) the magnification of (**c**); (**e**) the AA7075/Al-Cu2.3-Mg2.3-Zn6.6 weld; (**f**) the magnification of (**f**).

Figure 8 shows the phase evolution within FZ during the solidification process of the AA7075/Al-Cu_4.5_-Si_1.5_, AA7075/Al-Cu_1.5_-Si_4.5_ and AA7075/Al-Cu_2.3_-Mg_2.3_-Zn_6.6_ joints, respectively. It can be observed that, during the initial solidification stage, α-Al firstly crystallized from the liquid. Corresponding to the alloying elements, different secondary crystalline phases [19,20,21] such as Al_2_Cu, Al_2_CuMg, Al_5_Cu_2_Mg_8_Si_6_, Mg_2_Si, MgZn_2_ and Si form at the ending stage of solidification. This solidification path led to FZ presenting a dendrite-like microstructure with alloying elements segregating at the inter-dendrite zone.

**Figure 9:**
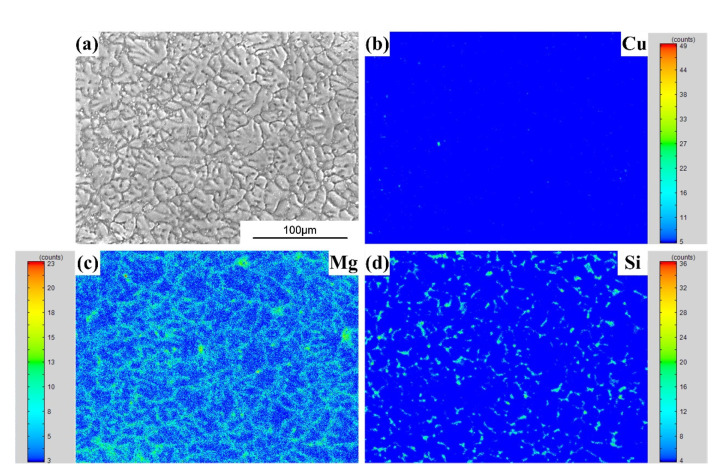
The EPMA mapping results (the AA7075/Al-Cu_1.5_-Si_4.5_ weld): (**a**) Secondary electronic image; (**b**) Cu distribution; (**c**) Mg distribution; (**d**) Si distribution.

**Figure 10:**
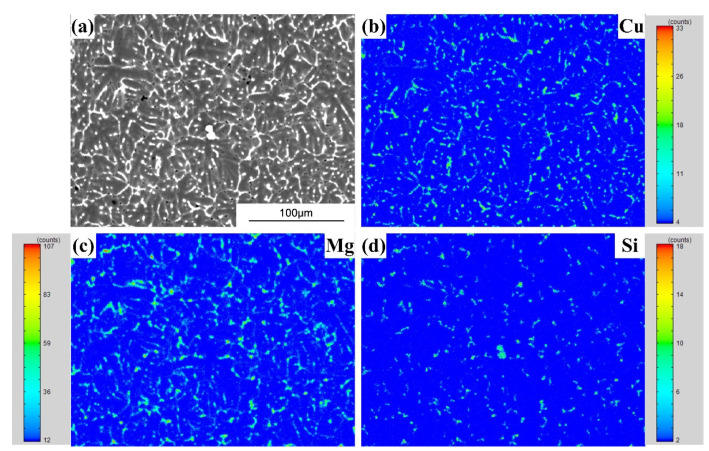
The EPMA mapping results (the AA7075/Al-Cu_4.5_-Si_1.5_ weld): (**a**) Secondary electronic image; (**b**) Cu distribution; (**c**) Mg distribution; (**d**) Si distribution.

**Figure 11:**
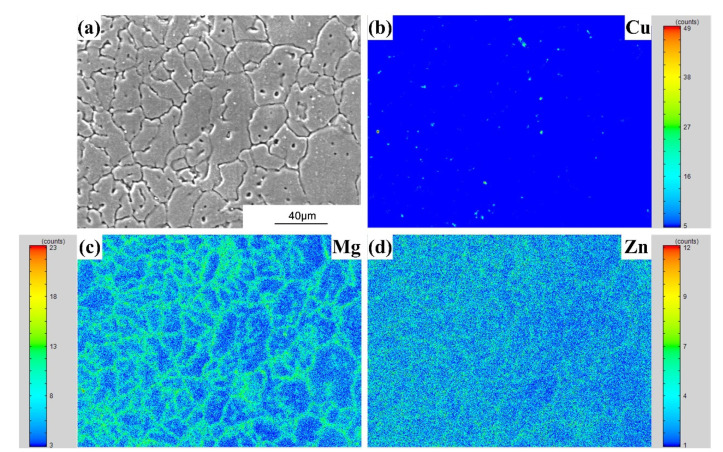
The EPMA mapping results (the AA7075/Al-Cu_2.3_-Mg_2.3_-Zn_6.6_ weld): (**a**) Secondary electronic image; (**b**) Cu distribution; (**c**) Mg distribution; (**d**) Si distribution.

FZ consists of a typical dendrite-like microstructure. Figure 9, Figure 10 and Figure 11 show the alloying element distributions of typical welds tested by EPMA. It can be observed that Cu, Mg, Si or Zn elements are segregated at the inter-dendrite zone, which supports the analysis result in Figure 8.

### 3.3. Mechanical Property of the Joints

The tensile test results are listed in Table 3. The data dispersion of the joints with the ER5356 filler wire occurs at a high degree. This is because liquation cracking exists within the joints. A previous work by the authors shows that the cracks within PMZ can increase the data dispersion of mechanical tests [22]. The fracture location shows that the joints with high liquation cracking susceptibility are prone to fracture in PMZ.

For all joints in the as-welded state, ductility is poor. This is because the alloying elements are segregated at the inter-granular zone, which forms a large number of IMCs. Besides, the fracturing location for joints with the ER5356 filler wire is the FZ/PMZ interface, while the other joints are fractured within FZ.

**Figure 12:**
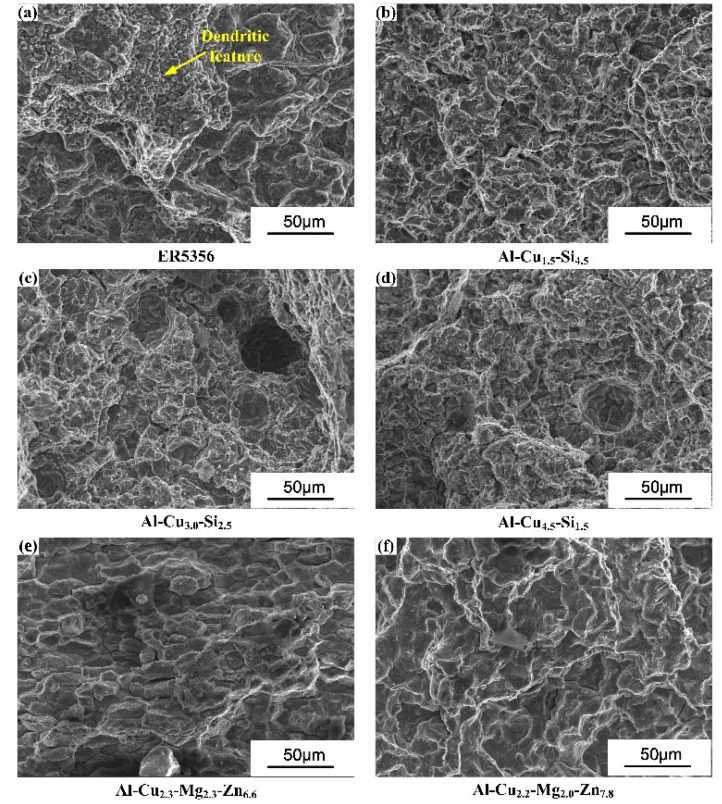
Fracturing surface: (**a**) The AA7075/ER5356 joint; (**b**) AA7075/Al-Cu_1.5_-Si_4.5_ joint; (**c**) the AA7075/Al-Cu_3.0_-Si_2.5_ joint; (**d**) the AA7075/Al-Cu_4.5_-Si_1.5_ joint; (**e**) the AA7075/Al-Cu_2.3_-Mg_2.3_-Zn_6.6_ joint; (**f**) the AA7075/Al-Cu_2.2_-Mg_2.0_-Zn_7.8_ joint.

Figure 12 shows the fracturing surface of each sample. The dendritic feature on the fracturing surface suggests the liquation cracking takes place at the FZ/PMZ interface of the joint with the ER5356 filler wire.

## 4. Conclusions

In this study, the liquation cracking susceptibility and mechanical properties of 7075 aluminum alloy GTAW joints with different filler wires were investigated. The phase evolution data of different joints were calculated using a multi-alloying-elements thermodynamics database to reveal the mechanisms. The following conclusions can be drawn:

(1) The effect of each alloying element on the solidification path was analyzed. The design principle of the Al-Cu-Si and Al-Cu-Mg-Zn filler wires in this work causes the *f_S_* of the weld to be a little higher than its counterpart of the workpiece under the specific temperature range.
(2) During the whole cooling period, the solid fraction within the fusion zone is higher than that of the partial melting zone for the joint with ER5356. By using Al-Cu-Si and Al-Cu-Mg-Zn filler metal, the solid fraction within the fusion zone is lower than that of the partial melting zone during the cooling period. By using Al-Cu-Si and Al-Cu-Mg-Zn filler metal, the liquation crack within the partial melting zone is restrained.
(3) The weld’s phase evolution data and the microstructure observation results indicate that α-Al firstly crystallized during the initial solidification stage to form the dendrite stem, while secondary crystalline phases at the inter-dendrite were generated during the ending solidification stage.
(4) The existence of liquation cracks led to a high degree of dispersion of the testing data in the joints’ tensile test. Compared to the joint with ER5356, the joint with Al-Cu-Si and Al-Cu-Mg-Zn filler metal exhibits a lower degree of data dispersion in the tensile test.

## Figures and Tables

**Table 1:** Chemical composition of filler and base metal (wt.%).

Filler Wire	Cu	Mg	Si	Zn	Al
ER5356	-	4.5	-	-	Bal.
Al-Cu_1.5_-Si_4.5_	1.5	-	4.5	-	Bal.
Al-Cu_3.0_-Si_2.5_	3	-	2.5	-	Bal.
Al-Cu_4.5_-Si_1.5_	4.5	-	1.5	-	Bal.
Al-Cu_2.3_-Mg_2.3_-Zn_6.6_	2.28	2.32	-	6.57	Bal.
Al-Cu_2.2_-Mg_2.0_-Zn_7.8_	2.15	2.08	-	7.81	Bal.

**Table 2:** Liquation cracking susceptibility and compositions of welds.

Filler Wire	Cracking Percentage	Cu in Weld (wt.%)	Mg in Weld (wt.%)	Si in Weld (wt.%)	Zn in Weld (wt.%)
ER5356	22.8%	0.6	3.58	0.16	2.2
Al-Cu_1.5_-Si_4.5_	8.3%	1.5	0.88	2.86	2.2
Al-Cu_3.0_-Si_2.5_	2.8%	2.4	0.88	1.66	2.2
Al-Cu_4.5_-Si_1.5_	2.8%	3.3	0.88	1.06	2.2
Al-Cu_2.3_-Mg_2.3_-Zn_6.6_	3.3%	1.89	2.13	0.22	6.14
Al-Cu_2.2_-Mg_2.0_-Zn_7.8_	1.4%	1.97	2.27	0.22	6.89

**Table 3:** Tensile test results.

Workpiece/Filler Wire	0.2% Yield Strength (MPa)	Average Tensile Strength (MPa)	Standard Deviation of Tensile Strength	Percentage Elongation	Standard Deviation of Percentage Elongation	Fracture Location
AA7075/ER5356	98.6	193.8	126.1	1.49%	1.17%	PMZ
AA7075/Al-Cu1.5-Si4.5	81.8	154.3	32.4	0.9%	0.2%	PMZ
AA7075/Al-Cu3.0-Si2.5	100.4	181.9	54.8	1.2%	0.3%	FZ
AA7075/Al-Cu4.5-Si1.5	157.8	286.8	13.5	2.4%	0.5%	PMZ
AA7075/Al-Cu2.3-Mg2.3-Zn6.6	153.8	279.4	35.1	2.2%	0.5%	FZ
AA7075/Al-Cu2.2-Mg2.0-Zn7.8	112.9	194.3	33.5	1.3%	0.2%	FZ

## Data Availability

Not applicable.
